# Supplementary material for: Patient safety in a rural sub-Saharan Africa hospital: A 7-year experience at the AIC Kijabe Hospital, Kenya
Source: PLOS Glob Public Health. 2024 Nov 12;4(11):e0003919. doi: 10.1371/journal.pgph.0003919 (PMC11556696; doi:10.1371/journal.pgph.0003919)
Supplement: S1 Data — (DOCX) [file pgph.0003919.s001.docx]

***HOSPITAL SURVEY ON PATIENT SAFETY***

**COMPILED BY:**

**Dr. Peter M Nthumba**

**Ms. Carol Mwangi**

**Mr. Moses Odhiambo**

**Patient Safety Survey Results for March 2019**

**Summary**

The data was collected using questionnaires administered to 370 hospital employees in all departments in the hospital. Response rate was 241 (65.1%) with129 (34.9%) not filling the questionnaires.

Majority 120 (50.0%) were Registered Nurses, working mostly in inpatient wards – 58 (24.1%). The overall Patient Safety Grade was acceptable at 53.5%. Most respondents were satisfied with the work climate provided by the administration (80.8%); most were satisfied that their suggestions were considered by their supervisors (74.2%).

47.2% felt they had enough staff to handle the workload, while 39.8% saying they needed more staff. 92.9% of respondents indicated that they are working towards improving patient safety at all times. 49.7% indicated that they worked longer hours than is best for patient care.

**RESPONSE RATE - (2013-2019)**

***Figure 1: response rate***

***Table 1: Patient Safety by Service Providers/Clinician in Percentage***

|  | Response | 2013  (N=350) | 2015  (N=307) | 2017  (N=281) | 2019  (N=241) | Overall |
| --- | --- | --- | --- | --- | --- | --- |
| Enough staff | Strongly Agree  Agree  Neither  Disagree  Strongly Disagree | 10.0  25.0  19.0  28.0  18.0 | 9.7  31.0  14.0  31.0  14.3 | 15.0  30.0  20.0  20.0  15.0 | 10.0  37.2  13.0  29.3  10.5 | **11.3**  **30.8**  **16.5**  **26.9**  **14.5** |
| Working longer hours than is best for Patient care. | Strongly Agree  Agree  Neither  Disagree  Strongly Disagree | 10.0  25.0  19.0  28.0  18.0 | 9.7  30.3  13.7  31.9  14.4 | 15.0  24.0  18.0  26.0  17.0 | 21.1  28.6  20.4  27.7  2.2 | **14.0**  **27.0**  **17.7**  **28.4**  **12.9** |

***Table 2: Patient safety at Various Departments in Percentage***

|  | Response | 2013  (N=350) | 2015  (N=307) | 2017  (N=281) | 2019  (N=241) | Overall |
| --- | --- | --- | --- | --- | --- | --- |
| Patients safety improving | Strongly Agree  Agree  Neither  Disagree  Strongly Disagree | 19.0  33.0  19.0  15.0  14.0 | 25.3  35.0  20.0  16.0  3.7 | 30.0  23.0  18.0  10.0  19.0 | 38.3  54.6  3.8  2.1  1.2 | **28.1**  **36.4**  **15.2**  **10.8**  **9.5** |
| Staff feel Blamed | Strongly Agree  Agree  Neither  Disagree  Strongly Disagree | 19.0  33.0  19.0  15.0  14.0 | 25.1  35.0  20.0  16.0  3.9 | 30.0  24.0  18.0  10.0  18.0 | 30.0  24.0  18.0  10.0  18.0 | **26.0**  **29.0**  **18.7**  **12.8 13.5** |
| Mistakes led to positive change | Strongly Agree  Agree  Neither  Disagree  Strongly Disagree | 11.0  39.0  24.0  17.0  9.0 | 27.0  35.0  20.0  15.0  3.0 | 24.0  30.0  10.0  14.0  22.0 | 13.1  55.7  18.1  11.0  2.1 | **18.7**  **40.0**  **18.0**  **14.3**  **9.0** |
| Adverse Events | Strongly Agree  Agree  Neither  Disagree  Strongly Disagree | 10.0  27.0  19.0  26.0  18.0 | 9.7  30.5  15.0  30.3  14.5 | 12.0  32.0  10.0  24.0  22.0 | 18.7  29.6  15.7  27.3  8.7 | **12.6**  **29.8**  **14.9**  **26.9**  **15.8** |
| We have safety problems | Strongly Agree  Agree  Neither  Disagree  Strongly Disagree | 10.0  26.0  19.0  27.0  18.0 | 9.7  30.3  14.0  31.9  14.1 | 14.0  35.0  20.0  15.0  16.0 | 7.2  23.3  18.1  40.0  11.4 | **10.2**  **28.6**  **17.8**  **28.5**  **14.9** |

***Table 3: Patient safety on Various Issues in Percentage***

|  | Response | 2013  (N=350) | 2015  (N=307) | 2017  (N=281) | 2019  (N=241) | Overall |
| --- | --- | --- | --- | --- | --- | --- |
| Patient safety Grade | Excellent  Very Good  Acceptable  Poor  Failing | 24.0  26.0  25.0  20.0  5.0 | 26.0  30.0  30.0  9.0  5.0 | 30.0  34.0  20.0  8.0  8.0 | 13.9  29.5  30.0  22.8  3.8 | **23.5**  **29.9**  **26.2**  **14.9**  **5.5** |
| Events Reported | No events  1 to 2 events reports  3 to 5 events reports  6 to 10 events reports  11 to 20 events reports  21 event reports or more | 53.0  22.0  18.0  4.3  1.4  1.3 | 68.4  16.0  7.5  4.6  1.9  1.6 | 65.0  10.0  16.0  3.0  2.0  4.0 | 61.5  25.5  4.8  2.9  3.9  1.4 | **61.9**  **18.4**  **11.6**  **3.7**  **2.3**  **2.1** |
| Years worked in Hospital | Less than 1 Year  1 to 5 Years  6 to 10 Years  11 to 15 Years  16 to 20 Years  21 years or more | 20.6  53.4  11.1  8.3  3.7  2.9 | 38.8  38.8  12.4  4.9  3.1  2.0 | 35.0  30.0  10.0  8.0  10.0  7.0 | 17.1  50.6  14.1  9.1  3.7  5.4 | **27.9**  **43.2**  **11.9**  **7.6**  **5.1**  **4.3** |
| Working Hours per week | Less than 20 hours  20 to 39 hours  40 to 59 hours  60 to 79 hours  80 to 99 hours  100 or more hours | *Missing | 2.0  3.3  87.2  2.3  3.9  1.3 | 4.0  5.0  80.0  4.0  5.0  2.0 | 2.1  3.9  83.7  7.3  2.6  0.4 | **2.7**  **4.1**  **83.6**  **4.5**  **3.8**  **1.3** |
| Mistakes caught and corrected before affecting patients | Always  Most of the time  Sometime  Rarely  Never | 24.0  26.0  25.0  20.0  5.0 | 26.0  30.0  30.0  9.0  5.0 | 30.0  34.0  20.0  8.0  8.0 | 13.9  29.5  30.0  22.8  3.8 | **23.5**  **29.9**  **26.2**  **15.0**  **5.4** |
| We Discuss ways to prevent errors from happening again | Always  Most of the time  Sometime  Rarely  Never | 1.8  33.6  26.6  28.0  1.8 | 29.0  33.0  24.4  8.7  5.2 | 12.0  10.0  22.0  30.0  26.0 | 1.7  5.0  25.3  33.6  34.4 | **12.0**  **20.5**  **24.6**  **25.9**  **17.0** |

**There is no common denominator due to missing data on ‘working hours per week’.*

***Table 4: Patient Safety by Service Providers/Clinician in Percentage***

|  | Response | 2013  (N=350) | 2015  (N=307) | 2017  (N=281) | 2019  (N=241) | Overall |
| --- | --- | --- | --- | --- | --- | --- |
| Supervisor consider suggestion | Strongly Agree  Agree  Neither  Disagree  Strongly Disagree | 24.0  27.0  19.0  26.0  4.0 | 28.0  28.0  10.0  27.0  7.0 | 30.0  25.0  20.0  20.0  5.0 | 20.0  54.2  12.1  7.0  6.7 | **26.0**  **34.3**  **14.0**  **20.0**  **5.7** |
| Work climate | Strongly Agree  Agree  Neither  Disagree  Strongly Disagree | 6.0  56.9  17.1  20.0  0.0 | 3.1  59.6  13.7  23.6  0.0 | 5.0  60.0  10.0  25.0  0.0 | 12.9  67.9  12.5  2.5  4.2 | **6.8**  **61.1**  **13.3**  **17.7**  **1.1** |
| Respondents position in the Hospital | Registered nurse  Clinical Officers  Doctors  Pharmacist  Ward secretary  others | 44.0  2.0  11.0  2.0  1.0  40.0 | 56.0  3.1  4.3  8.0  0.6  28.0 | 60.0  3.0  8.0  5.0  2.0  22.0 | 50.0  7.5  6.3  5.9  0.8  29.5 | **52.5**  **3.9**  **7.4**  **5.2**  **1.1**  **29.9** |

**Figure Representation of Patient Safety Comparison from 2013 to 2019**

***Figure 1: primary work area/unit of respondent’s***

1. **YOU HAVE ENOUGH STAFF TO HANDLE THE WORKLOAD**

***Figure 2: staffs Feedback on number of staffs to handle workload***

1. **STAFF IN THIS UNIT WORK LONGER HOURS THAN IS BEST FOR BEST FOR PATIENT.**

***Figure 3: working longer hours than is best for patient***

1. **What is your staff position?**

*Figure 4: staff position*

1. **STAFF FEEL LIKE THEY ARE BLAMED FOR THEIR MISTAKES**

*Figure 5: staffs blamed*

1. **WE HAVE PATIENT SAFETY PROBLEMS IN THIS UNIT**

*Figure 6: Having patient safety problems in units.*

1. **MY SUPERVISOR SERIOSLY CONSIDERS STAFF SUGGESTIONS FOR IMPROVING PATIENT SAFETY.**

*Figure 7: supervisor considering staff suggestion.*

1. **WHEN AN ADVERSE EVENT OCCURS, USUALLY MORE FOCUS IS PLACED ON THE PERSONAL ERROR THAN THE UNDERLYING PROBLEM.**

*Figure 8: focus on errors than the underlying problem*

1. **STAFF IN THIS UNIT ARE ACTIVELY DOING THINGS TO IMPROVE PATIENT SAFETY.**

***Figure 9: units actively working to improve patient safety***

1. **MISTAKES HAVE LED TO POSITIVE CHANGES**

***Figure 10: mistakes have led to positive changes***

1. **IN THIS UNIT WE DISCUS WAYS TO PREVENT ERORS FROM HAPPENING AGAIN.**

***Figure 11: discussing ways to prevent errors from happening***

1. **WHEN A MISTAKE IS MADE, BUT IS CAUGHT AND CORRECTED BEFORE AFFECTING THE PATIENT, HOW OFTEN IS THIS REPORTED**

***Figure 12: reporting of mistakes corrected before affecting patients***

1. **HOSPITAL MANAGEMENT PROVIDES WORK CLIMATE THAT PROMOTES PATIENT SAFETY.**

***Figure 13: work climate that promotes patient safety***

1. **TYPICALLY, HOW MANY HOURS PER WEEK DO YOU WORK IN THIS HOSPITAL?**

***Figure 14: staff working hours per week***

1. **HOW MANY EVENTS REPORTS HAVE YOU FILLED**

***Figure 15: number of Event report filed***

1. **HOW LONG HAVE YOU WORKED IN THE HOSPITAL**

***Figure 16: respondents’ years of service in this hospital***

1. **PATIENT SAFETY GRADE**

***Figure 17: Patient safety grade***


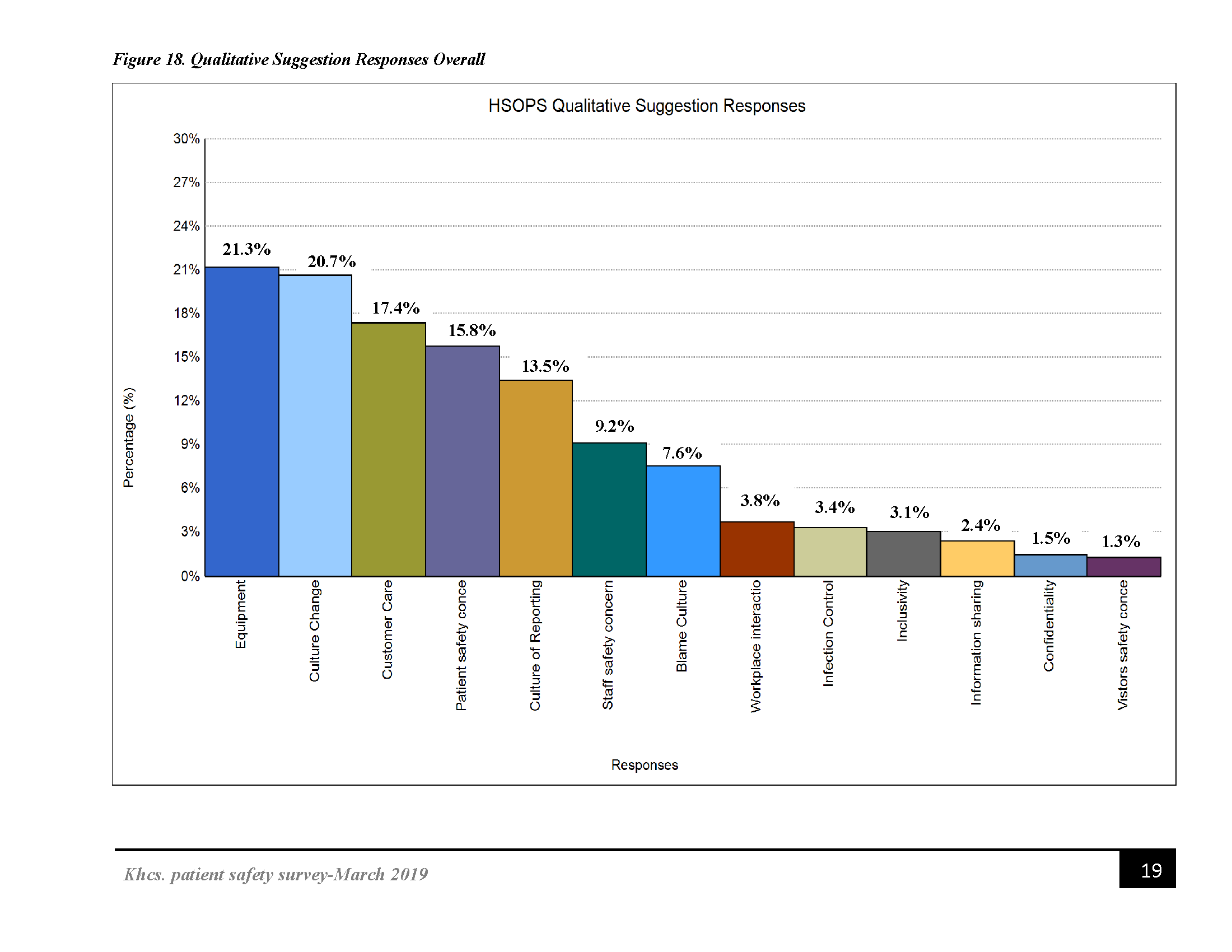


**Table 3. Major concerns and recommendations**

| **Themes** | **Major Discussion Points** |
| --- | --- |
| **Blame Culture** | - Management should avoid blame games when errors occur - When an error occurs it should be analysed so that we can learn from it |
| **Culture Change** | - Staff have become more responsible after culture change training - Management has provided good climate for patient safety; patient safety is a priority at all times - Medical error data collection tool adopted has helped improve patient safety in pharmacy |
| **Culture of Reporting Incidents** | - Encourage culture of reporting incidents even minor one; review to learn from them. - Adopted honest medical legal policy to tell the truth about any error occurring to any patient - Frequent Customer Due Diligence with regards to patient safety is recommended for the purpose of recognition, documentation, and prevention, with prompt measures to taken |
| **Customer Care** | - There should be someone to direct patients and relatives to different departments within the hospital - *****Waiting areas are congested, waiting times are too long, paperwork should be done before theatre - *****Washrooms are inadequate; **†**treatment rooms at physiotherapy department are very small and poorly ventilated for patients and care givers - ***†**Uncovered pathway towards private clinic/palliative makes patient very vulnerable when it’s raining, slippery floors should be changed, especially in hallways |
| **Equipment** | - Hospital beds lack rails and proper patient restraints; wheelchairs, stretchers, trolleys, and exam couches need attention - *****X-ray lead gowns are needed for patient protection; - *****Provide back friendly seats; warm bathing water, and heaters for very cold wards/rooms to ensure patients are comfortable at night. |
| **Inclusivity** | - Involve care givers on the ground before putting measures patient safety - There is need for consultation on materials to be bought for use by care givers |
| **Infection Control** | - Equip security officers with modern security equipment e.g. door scanners to reduce body contact - Uncovered and stinking drainage under Customer Care office |
| **Information Sharing** | - Communication systems should be improved - Prompt survey feed back - Make available standard policy documents at all times. |
| **Professionalism** | |
| **Confidentiality** | - Let’s be more responsible and maintain high level of confidentially |
| **Workplace Interaction** | - Respect between staff and managers should be improve - Co-operation between departments should be improved - Need for better system for handing over and picking critical issues on our patient (Handing over SOPs) |
| **Safety** | |
| **Patient Safety* Concerns** | - Have drill to prepare all staff and patient on patient safety - Emergency exit including stirs in private ward should be improved - Need for better system for handing over and picking critical issues on our patient. |
| **Staff Safety Concerns†** | - Have drill to prepare all staff and patient on patient safety - Fire exit is need at physiotherapy department - Emergency exit including stairs in private ward should be improved - Provide adjustable beds with rails for patients at BKKH some are too low hence back pains to staffs |
| **Visitor’s Safety Concerns** | - Slippery floors especially on the hallways should be changed |
