# Supplementary material for: Patient safety in a rural sub-Saharan Africa hospital: A 7-year experience at the AIC Kijabe Hospital, Kenya
Source: PLOS Glob Public Health. 2024 Nov 12;4(11):e0003919. doi: 10.1371/journal.pgph.0003919 (PMC11556696; doi:10.1371/journal.pgph.0003919)
Supplement: Attachment — Submitted filename: PLOS Kenya Safety Culture.docx [file pgph.0003919.s002.docx]

Well informed/written and comprehensive overview of patient safety culture in Africa, melded with primary data from the local hospital culture surveys over a seven-year period. Somewhat unusual format, but perhaps it “works” here.

The literature review seems basic and perhaps not systematic but generally informative and relevant. I have not re-searched the topic to closely examine whether they have picked up all the relevant papers, but most of what they've included seems good and germane.

I question the title--whether it is exactly appropriate for the article contained here. The "high turnover" seems to come a bit out of the blue, although we know this is a very important patient safety issue and seems especially true in this hospital. (tho we have nothing to compare it to). And the word "developing" also doesn't seem to fit with this paper which does little to describe or study what initiatives are being taken to “develop” such a culture. On the contrary it is really a reporting of the survey responses over time, which as the authors point out, is a innovative contribution in itself. Thus t the authors shouldn't use the title to claim something there specific study did not actually do.

A related problem is that someone could read this as a promotional cheerleading piece that selectively pulls out areas where there is bona fide improvement and touts them. Since the authors were involved in both overseeing quality initiatives in the hospital as well as conducting the survey and crafting the language here, I think they should go through the paper with a I on softening any cheerleading and promotional commentaries. Perhaps there is even a conflict of interest that needs to be acknowledged in the discussion. Better to let the "facts" speak for themselves --and there are a number of impressive areas of improvement particularly in the 2019 survey.

This brings us to the other concern about the paper in general. The improvements and findings are a hodgepodge of some areas getting better, others getting worse or staying the same, and others going up and down over time. It is somewhat helpful that they are providing context to perhaps explain some of these results (e.g. revolt of the staff about leadership and working conditions), but I think the authors should again carefully go through the paper and minimize attributions to these changing numbers, certainly highlighting that these are at best hypotheses and associations rather than conclusions that can be drawn from the data.

Of course when we think about staff turnover and chaos in culture, the last three years are even more so, and would be interesting to see how that impacted on these metrics. Did the authors do a survey in 2021? Perhaps not and thus it wouldn't be fair to expect that they would if the hospital was in the throes of the COVID crisis.

The authors mention a relatively high response rate although the numbers of responses are only in the several hundred. Is this in fact the total number of staff in the surveyed categories? A staffing number of 650 is mentioned but it appears that the denominator for this study is something smaller. There seems to be an error/typo in the text when you discuss staff not generally in direct contact with patients – these groups of staff excluded? If so that needs to be stated.

The authors mention "six champions who ran the day-to-day activities of the project." What exactly is "the project." I suspect they are referring to the quality and safety unit of the hospital but these descriptors are not well enough defined here.

The qualitative comments thematic analysis seems a tad week. I would've hoped that the authors could've solicited more substantive and critical comments from those surveyed. Instead might this be related (for example) to the higher response rate where workers were required to fill this out but many did so perfunctory. The responses seem mostly short and more platitudes than critical insights.

Staff perceptions of hospital management support for patient safety rose from 62% to 80% – this and several other big jumps struck me as both impressive as well as potential red flags. Could there be unmeasured confounders or artifacts in what we are seeing here. These are obviously different employees (given the high turnover rate) but what about the mix of employees and the fact that this doesn't correlate with several other measures in the same survey.

“it is apparent from the study that the institutional working environment is an important determinant of patient safety perception by staff." I am not sure that the authors can justify concluding that there paper proves this. i.e. more of a correlation and a hypothesis, not necessarily something we can draw hard conclusions about here. .

The installation of an EMR must have been a huge confounder (and perhaps could be one factor to explain some of the dramatic changes in 2019).

In conclusions I would say it's the longest "longitudinal" institutional HS OPS data.

Figure 1 needs to be improved so the image is less blurry. I had trouble reading it.

Also for the editors, when I downloaded the PDF, the tables were not accessible when I clicked under supplemental material. Please fix this problem in editorial manager as I assume you want to make it easier for your reviewers. (Normally the PDF that is generated includes the tables from the paper).
