# Supplementary material for: Patient safety in a rural sub-Saharan Africa hospital: A 7-year experience at the AIC Kijabe Hospital, Kenya
Source: PLOS Glob Public Health. 2024 Nov 12;4(11):e0003919. doi: 10.1371/journal.pgph.0003919 (PMC11556696; doi:10.1371/journal.pgph.0003919)
Supplement: Attachment — Submitted filename: Reviewer REsponse.docx [file pgph.0003919.s003.docx]

|  | **Reviewers’ Comments** | **Responses to Reviewer’s Comments/ Questions** |  |
| --- | --- | --- | --- |
|  | **Journal Editor** | | |
| 1 | <https://www.afro.who.int/sites/default/files/2020-10/Ghana%20Annual%20report%202019.pdf> | Reference added to similar reference to the same event | Reference added |
| 2 | <https://pso.ahrq.gov/sites/default/files/wysiwyg/npsdpatient-safety-culture-brief.pdf> | Reference added to similar reference to same material (by AHRQ) | Reference added |
| 3 | <https://www.emerald.com/insight/content/doi/10.1108/IJHCQA-07-2012-0072/full/html> | Reference added to previous reference to a different publication | Reference added |
|  |  |  |  |
| 4 | <https://journals.lww.com/md-journal/Fulltext/2018/09210/Effectiveness_and_limitations_of_an.112.aspx> | Ramirez et al report on the use of an incident reporting system (IRS), using the number of reported adverse events as a surrogate measure of efficacy of the IRS. Our study focuses on staff perception of the hospital safety culture, and uses the HSOPS tool to report this. The only point of intersection between this and our work is the focus on patient safety. | Reference not included |
| 5. | In the online submission form, you indicated that "Data availability statement: Data are available upon reasonable request. Please contact the corresponding author to discuss the feasibility of obtaining access to a deidentified data set". All PLOS journals now require all data underlying the findings described in their manuscript to be freely available to other researchers, either 1. In a public repository, 2. Within the manuscript itself, or 3. Uploaded as supplementary information.  This policy applies to all data except where public deposition would breach compliance with the protocol approved by your research ethics board. If your data cannot be made publicly available for ethical or legal reasons (e.g., public availability would compromise patient privacy), please explain your reasons by return email and your exemption request will be escalated to the editor for approval. Your exemption request will be handled independently and will not hold up the peer review process, but will need to be resolved should your manuscript be accepted for publication. One of the Editorial team will then be in touch if there are any issues. | Data will be available as supplementary information | Summated data is provided |
| **Reviewer 1** | | | |
| 1 | Title:  The "high turnover" seems to come a bit out of the blue, although we know this is a very important patient safety issue and seems especially true in this hospital. (tho we have nothing to compare it to). And the word "developing" also doesn't seem to fit with this paper which does little to describe or study what initiatives are being taken to “develop” such a culture. On the contrary it is really a reporting of the survey responses over time, which as the authors point out, is a innovative contribution in itself. Thus t the authors shouldn't use the title to claim something there specific study did not actually do. | We are grateful for the reviewer’s feedback, important to improving the quality of our manuscript. Thank you for the time taken to critically read through this work. We believe that it will help our region break into a new era of patient safety in our healthcare institutions, a badly needed ingredient in our systems.  We have changed the title | Patient safety in a rural sub-Sahara Hospital: A 7-year experience at the AIC Kijabe Hospital, Kenya. |
| 2 | Literature review: The literature review seems basic and perhaps not systematic but generally informative and relevant. I have not re-searched the topic to closely examine whether they have picked up all the relevant papers, but most of what they've included seems good and germane. | As indicated in the text, patient safety, and safety culture, is a fairly new concept within sub-Saharan Africa. The first Africa Patient Safety Day was held in 2019. There is therefore very little written on this subject in the sub-continent. This therefore, is a narrative review of the subject. | No changes |
| 3 | A related problem is that someone could read this as a promotional cheerleading piece that selectively pulls out areas where there is bona fide improvement and touts them. Since the authors were involved in both overseeing quality initiatives in the hospital as well as conducting the survey and crafting the language here, I think they should go through the paper with a I on softening any cheerleading and promotional commentaries. Perhaps there is even a conflict of interest that needs to be acknowledged in the discussion. Better to let the "facts" speak for themselves --and there are a number of impressive areas of improvement particularly in the 2019 survey. | On promotional cheerleading, we would like to disagree with the reviewer: We make this bold statement: “**KH leadership perception of staff satisfaction with their working conditions, as well as the explanation for the high staff turnover, was misleading.**”  I believe we have as objectively as possible attempted to interpret the results obtained, giving possible explanations for phenomena observed.  We would be most grateful to the reviewer if they highlighted areas where positive statements do not reflect the results obtained.  Additionally, prospective surveys brought out issues that needed changing – these were reported to senior management who then prioritized them. | We have added the statement under Declaration: ‘The authors were members of the Surgical Unit Safety Program (SUSP) core team; they participated in the implementation and evaluation of SUSP, including its perceived impact on the institutional safety culture.’  We have ‘toned down’ some of the data interpretations to a more neutral stance:  The differences in the mean percent positive dimension scores across most domains of HSOPS in 2019 (Table 2a), are likely the result of cumulative efforts of the KH leadership to engage with all cadres of staff.  All the HSOPS results were reported to, and discussed with senior management; the senior management addressed some of issues that came out of each report. |
| 4 | This brings us to the other concern about the paper in general. The improvements and findings are a hodgepodge of some areas getting better, others getting worse or staying the same, and others going up and down over time. It is somewhat helpful that they are providing context to perhaps explain some of these results (e.g. revolt of the staff about leadership and working conditions), but I think the authors should again carefully go through the paper and minimize attributions to these changing numbers, certainly highlighting that these are at best hypotheses and associations rather than conclusions that can be drawn from the data. | We have deleted some of the sentences with explanations of the results obtained.  We have added a statement explaining the staff revolt. | ~~The drop in 2019 is difficult to explain, as all other domain scores improved (Table 2a). The staff perception of the overall institutional patient safety was lowest at 36% in 2017; curiously though, 64% of staff scored patient safety grade at excellent or very good in the same year - this improvement in patient safety grade was statistically significant (p=0.004), (Table 2a)~~.  Staff unrest because of what was reported to be an insensitive leadership, with difficult working conditions, demanded for, and led to a radical leadership change |
| 5 | Of course when we think about staff turnover and chaos in culture, the last three years are even more so, and would be interesting to see how that impacted on these metrics. Did the authors do a survey in 2021? Perhaps not and thus it wouldn't be fair to expect that they would if the hospital was in the throes of the COVID crisis. | In 2021, we only ran a survey in the Maternity Department, and so could not include the results in this paper | No change |
| 6 | The authors mention a relatively high response rate although the numbers of responses are only in the several hundred. Is this in fact the total number of staff in the surveyed categories? A staffing number of 650 is mentioned but it appears that the denominator for this study is something smaller. There seems to be an error/typo in the text when you discuss staff not generally in direct contact with patients – these groups of staff excluded? If so that needs to be stated. | The reporting of response rates is calculated using a standardized formula. The numerator is the number of surveys returned, while the denominator is the number of eligible staff who received a survey. Given the workings of a hospital, it not possible to at any time capture 100% of the workers, and therefore, the denominator changed with each survey, because different numbers were recruited each time. This was based on the number of surveys distributed (denominator) versus the number returned (numerator). There were no exclusions in the analysis, since patient safety is a concern for every person working in the hospital. E.g., Slippery floors is the responsibility of Housekeeping – if a patient slips and falls, that is a patient safety issue. | No change |
| 7 | The authors mention "six champions who ran the day-to-day activities of the project." What exactly is "the project." I suspect they are referring to the quality and safety unit of the hospital but these descriptors are not well enough defined here. | The WHO African SUSP project is introduced at the tail end of the introduction. This project was run by a core team of 6 patient safety champions.  We have added the text ‘…the WHO African SUSP project’ to clarify this. In addition, we have included a reference to a publication that explains the project, and its execution. | A SUSP core team comprising of six patient safety champions ran the day-to-day activities of the WHO African SUSP project |
| 8 | The qualitative comments thematic analysis seems a tad week. I would've hoped that the authors could've solicited more substantive and critical comments from those surveyed. Instead might this be related (for example) to the higher response rate where workers were required to fill this out but many did so perfunctory. The responses seem mostly short and more platitudes than critical insights. | We are in agreement with the reviewer. We would certainly have preferred richer responses. However, we used the HSOPS tool, which has a small area for free comments. We analyzed all the comments made. While we did encourage staff to feel free and consider patient safety issues while responding, in the end, this is all we had. | No changes made |
| 9 | Staff perceptions of hospital management support for patient safety rose from 62% to 80% – this and several other big jumps struck me as both impressive as well as potential red flags. Could there be unmeasured confounders or artifacts in what we are seeing here. These are obviously different employees (given the high turnover rate) but what about the mix of employees and the fact that this doesn't correlate with several other measures in the same survey. | Some of the results noted are difficult to tease out and explain. While the high turnover is certainly a pointer to potential bias, we have found no potential explanations for the apparent aligning of all the outcome variables. We therefore have reported the data as analysed. We will make the data publicly available for scrutiny | Data will be availed for public scrutiny |
| 10 | “it is apparent from the study that the institutional working environment is an important determinant of patient safety perception by staff." I am not sure that the authors can justify concluding that there paper proves this. i.e. more of a correlation and a hypothesis, not necessarily something we can draw hard conclusions about here. . | We have changed previous statements of ‘fact’ not proved by the study into hypotheses. | From the results of this study, the authors hypothesize …. |
| 11 | The installation of an EMR must have been a huge confounder (and perhaps could be one factor to explain some of the dramatic changes in 2019). | Initially, the EMR (and somewhat to date) was received with disdain, as we started of with an unstable system that 4 years later continues to be tweaked. Although there was a campaign to popularize it, we would have expected it to negatively affect the perception of patient safety, given the initial experiences that included disappearing notes etc. | No changes |
| 12 | In conclusions I would say it's the longest "longitudinal" institutional HS OPS data. | We are grateful for the reviewer’s suggestions | ‘longitudinal’ added |
| 13 | Figure 1 needs to be improved so the image is less blurry. I had trouble reading it. | Thank you for this suggestion | Figure manipulated to improve quality |
| 14 | Also for the editors, when I downloaded the PDF, the tables were not accessible when I clicked under supplemental material. Please fix this problem in editorial manager as I assume you want to make it easier for your reviewers. (Normally the PDF that is generated includes the tables from the paper). | Directed at the Editorial team | No changes |
