# Supplementary material for: Patient safety in a rural sub-Saharan Africa hospital: A 7-year experience at the AIC Kijabe Hospital, Kenya
Source: PLOS Glob Public Health. 2024 Nov 12;4(11):e0003919. doi: 10.1371/journal.pgph.0003919 (PMC11556696; doi:10.1371/journal.pgph.0003919)
Supplement: Attachment — Submitted filename: Response to Reviewer Comments.docx [file pgph.0003919.s004.docx]

| The authors would like to truly appreciate the reviewers for a very thorough review. Thank you for taking your time to help us improve this manuscript. | | |
| --- | --- | --- |
| **Reviewer Comment** | **Author Comment** | **Manuscript Revisions** |
| ***Reviewer 1:*** |  |  |
| Mostly responsive revisions. No other changes at this point. Some of the prose could use a once over by a prose editor | Thank you for these comments we have carefully reviewed the manuscript and corrected its prose. | Extensive prose adjustments |
| ***Reviewer 2:*** |  |  |
| Thankyou for giving me this opportunity to review the research article. It was my pleasure to think upon the innovative ways in which the article can be enhanced more profoundly.  The manuscript is a good in-depth study of patient safety culture at AIC Kijabe Hospital, providing unique insights into the evolution and challenges of building a safety culture in sub-Saharan African healthcare institutions. | The authors thank the review for a thorough review of our manuscript. We will make a detailed response to the queries posed. | No changes |
| ABSTRACT:  1. The Methods section lacks details on how the HSOPS tool was applied, including sample size, demographics, any modifications made to the tool to adapt it with the hospital needs. | We have made changes to address the shortcomingsg identified. | … and also report their experience using the HSOPS tool in Kijabe Hospital, with 650 employees, over a 7-year period. The HSOPS tool, with no local modifications, was distributed to all employees during each survey. |
| 2. The Results section needs a more specific information on magnitude of these improvements or any other trends observed over the study period. | Thank you for this feedback. We have made specific information on the improvements mentioned. | The average response rate during the study period was 84.5% (range 65.1% to 93.6%). In general, the mean percent positive dimension scores of most domains improved in the 2019 survey (p<0.05), including reduced staff turnover, improved hospital support for patient safety (p<0.0001), amongst other domains. The overall patient safety grade (excellent/very good) was 50% (range 43 -64%). |
| 3. Although staff turnover and leadership changes are acknowledged as problems, the conclusion retains that sub-Saharan Africa fulfils patient safety objectives. But rather than specific proof from the study's findings, it hinges on hypothesized relationships. | We have restructured this section to reflect the study findings. | … embraced in sub-Sahara Africa. Patient safety champions, a generative institutional leadership that is supportive of patient safety, are important for the development of an institutional safety culture. … |
| INTRODUCTION: A thorough description of patient safety culture, its barriers, and efforts to enhance it—particularly in sub-Saharan Africa—are given in the introduction. Nevertheless, a few crucial details are absent: |  |  |
| 1. The specific objectives of the study are missing. The purpose of doing the study should be more emphasized upon. | “*The authors used the HSOPS tool to establish the baseline status of patient safety culture, to raise staff awareness about patient safety and thereafter, to examine trends in patient safety culture change over time, and to evaluate the institutional cultural impact of patient safety initiatives and interventions*” is a statement found at the end of the introduction which describes our objectives in doing this study.  We included an additional statement in order to further cement this | In this report, the authors describe their institutional patient safety trends over a seven-year period, through a review HSOPS results, and hope that their experiences will stimulate regional healthcare institutions to start a similar journey. |
| 2. There is no specific data of current patient safety culture in sub-Saharan Africa which may probably also serve as a baseline for the comparison of this study to the audience. | References 37 to 39 are the only patient safety reports comparable to our study. We have added the precise overall percentage patient safety perception for comparison.  We could not find other SSA reports for comparison. | patient safety perception (between 44% and 46%) was higher in our institution |
| METHODS:   1. How were the surveys administered to the participants? | This is explaned in the manuscript as follows, “*For each survey, the HSOPS tool was printed and given to departmental heads to distribute amongst team members: it was filled anonymously, with no participant identifiers*.”  We have added a statement to explain the fact that staff were prepared for the survey. | …. distributed, the SUSP core team met staff in their departments and taught them about the HSOPS tool, during these sessions, staff were … |
| Was there any modification done to the HSOPS tool? | We have added a statement to answer this question. | The HSOPS tool was used without any local modifications. |
| 2. Since the approach of the study design was narrative reviews, was there any potential bias in study selection and data synthesis? | The participants filled the HSOPS tool, which includes a space for free text. We only analyzed the data in the survey tool.  We did not perform any narrative reviews.  Because the study targeted all the hospital employees, there was no potential bias we could identify. | No change |
| 3. The methods outline how hospital staff  members are given the HSOPS tool after being given an overview of the tool and patient safety principles. Despite this, the methodology would be strengthened by providing information on the sampling strategy (such as convenience sampling or random sampling) and the steps taken to guarantee the sample's representativeness. Furthermore, even though using anonymous surveys aids in maintaining anonymity, it's very important to address any potential biases brought about by self-selection or non-response. | As indicated in response to question 2, we prepared adequate HSOPS questionnaires for all hospital employees. Although survey tool distribution was through departmental heads, it was impossible to determine the source of the questionnaires returned, or the characteristics of non-responders.  Staff not on duty throughout the survey period were most likely amongst non-responders, however, the authors had no way of identifying responders from non-responders. This was designed to protect staff from fear of being identified, as this would have itself created a bias in staff responses. | No changes |
| 4. Also, the participant recruitment information is missing. | We believe that this paragraph addresses participant recruitment. This includes some *statements* added in response to previous questions. (in red)  “*As part of the baseline SUSP study, the HSOPS tool was administered to all hospital employees in their different departments, after an explanation of the tool and data collection process. The HSOPS tool was used without any local modifications. For each survey, the HSOPS tool was printed and given to departmental heads to distribute amongst team members: it was filled anonymously, with no participant identifiers. In the week before the questionnaire was distributed, the SUSP core team met staff in their departments and taught them about the HSOPS tool, during these sessions, staff were taught the precepts of patient safety, and shown the African SUSP patient safety video*” | No additional changes |
| 5. The section majorly lacks the data analysis part which tells about the coding process in qualitative data. If there were any theoretical frameworks used for it. For the quantitative data, there is no mention about the specific statistical tests used. | We have edited the statements to clarify statistical analysis of both qualitative and quantitative data. | The team coded participants’ free text comments into broad categories as themes emerged. The final thematic analysis involved merging similar themes into broader categories. Thematic saturation was not anticipated because of the small number of participants that wrote free text comments.  Quantitative analysis was performed using STATA version 18 (StataCorp LLC, College Station, TX), and reported as descriptive statistics. |
| 6. Transparency and adherence to ethical standards are enhanced by the Institutional Ethical Review Board's ethical approval being mentioned. But there is no justification provided for not obtaining the formal consent from participants. This particularly raises ethical concerns regarding informed consents and the participants’ rights. | Thank you for this comment. The authors agree with them wholeheartedly.  The WHO SUSP study was run in 2013 – the HSOPS tool was part of the approved tools. However, even then, in order to avoid any possibility of identifying the respondents, individual consents were avoided. This was discussed with the participants at the time of preparing them for the survey (the week before each survey). Participants were encouraged to participate, but were also informed that withdrawal from participating would not attract any punitive measures. Because of the anonymity, staff were a lot freer to participate, knowing that they could not be identified. Thus participant autonomy was not breached.  We have made this statement under the ‘Erthics Statement’: “*In order to maintain anonymity, a formal consent for participant participation was not obtained*.” | No changes |
| RESULTS:  1. It would be helpful to include information on the distribution of respondents across different departments or units within the hospital to assess the representativeness of the sample. | Thank you for this comment. Because of anonymity, we relied on the partipants to report their job description. This is recored in Table 2b and explained in the ‘Results’ section under ‘Respondents position in the hospital’. Knowing their departments would have potentially identified them, which might have created a reporting bias in their responses. It was thus it was impossible to determine the source of the questionnaires returned, or the characteristics of non-responders, except what they reported. | No changes |
| 2. Additional details on the methods used to calculate turnover rates and perceptions of support would strengthen the analysis. | In response, we have included the following explanation | Because the total staff were 650 throughout this period, the number of staff reporting having worked ‘less than 1 year’, were newly employed within the previous 12 months; an increase in the percentage of staff who had worked ‘less than 1 year’, indicated an increase in the number of new staff, an indication of increased staff turnover. Conversely, a reduction in this category, indicated staff transitioning within the institution, to the ‘1 to 5 years’ category, a surrogate indicator for reduced staff turnover. |
| 3. Also, there can be an improvised detail on how was the missing data of 2013 was handled in the analysis to ensure validity of the results? | Thank you for this comment. We have explained how we handled missing data | Because the missing data affected a single category in 2013 (working hours per week), it did not affect data analysis; we did not perform any statistical comparisons in this category. |
| DISCUSSION:  1. The discussion emphasizes the necessity for validation through patient perceptions and experiences, accurately pointing out the limitations of depending only on staff opinions of patient safety culture. | Thank you – this is an important point, and is a limitation of our study. | No changes |
| In subsequent research, it would be beneficial to talk about possible methods for integrating patient input into the evaluation of patient safety culture. | We have included a statement in support of this concern. | Future studies measuring patient safety should include patient perception of safety; indeed a patient perception domain addition to the HSOPS tool should be considered, to enable patient validation of hospital staff perceptions, as well as empower patients in impacting safety initiatives in hospitals. |
| 2. A more thorough examination of the contextual differences between healthcare settings in high-income countries and those in low- and middle-income countries, particularly in sub-Saharan Africa, and how these differences may influence patient safety culture, would be beneficial, even though the section compares the findings with those of studies conducted in the USA. | Thank you for this feedback.  Although we used a USA-developed tool, our primary focus was to compare patient safety activities within sub-Saharan Africa. Therefore, we fill that a review of contextual differences between these two environments are outside the scope of our current discourse. | No change |
| 3. Furthermore, greater information on the precise steps taken and their effects on patient safety culture would be helpful, even if the discussion highlights the corrective measures that hospital management took in response to staff opinions and remarks. | We have added a column detailing changes effected as a result of staff feedback. | Please see table 3, under a new column “**Changes effected**” |
| CONCLUSION:  1. Since the study focused on a single institution, it would be beneficial to discuss the extent to which the findings may be applicable to the other healthcare settings in sub-Saharan Africa, considering variations in the organizational culture, resources and patient population. | Thank you for this feedback. Whereas, as we have indicated, we believe that our report clearly demonstrates that patient safety can (and should) be implemented and embraced in sub-Saharan Africa, a few nuances in our study make external validity of our results difficult to claim. We have made this statement under conclusions:  “*The authors excluded some HSOPS composite scores – although scaling down the number of composites is permitted as described in the HSOPS users guide.* | No changes |
| 2. Considering the study limitations, more details can be emphasized on how these limitations may have influenced the interpretation of the study findings or the generalizability of the results beyond AIC Kijabe Hospital. | Thank you for this comment. We have included the following as a response | While the above-mentioned limitations make generalizability outside KH difficult, we believe that the experiences reported in the process of developing a patient safety culture, and the low-resource utilization of the process, demonstrate principles that institutions in similar environments can use to successfully develop a safety culture. |
| ***Reviewer 3:*** |  |  |
| Summary of the research and overall impression  The authors apply an adapted version of the Hospital Survey on Patient Safety Culture (HSOPS) as a tool to assess and compare institutional safety culture over a 7-year period (2013-2019) at Kijabe Hospital, Kenya. Given the perfunctory nature of safety and quality studies in Low- and Middle-Income Countries (LMICs), including Sub-Saharan African countries, I appreciate the efforts of the authors to document the introduction and institutionalization of a safety culture in their hospital and report on that over time from 2013-2019. The authors predominantly discuss the positive results from the 2019 survey in relation to the 2013 survey and discuss how contextual factors could explain some of the 2017 survey results. | Thank you for appreciating our work. | No changes |
| Discussing qualitative data about institutionalizing a safety culture could add considerable value to the study as institutionalizing a safety culture is difficult to be captured solely via a survey. It could be very valuable to know how safety measures improved and the hospital activities that that entailed between survey periods. | Thank you for this observation. We have added a column detailing changes effected as a result of staff feedback. | Please see table 3, under a new column “**Changes effected**” |
| It takes effort, activities, and communication to build and institutionalize a safety culture and the paper does not contribute to that process of construction and institutionalization of the safety culture over time. | While agreeing that the establishment of a safety culture is a long journey, we wish to differ with the reviewer, as we have reported the following:   1. Table 3 reports a number of safety initiatives undertaken to enhance patient safety .   We have reported the establishment of the Quality Improvement Department, a funded, staffed department that oversees issues that include Patient Safety.   2. “*Furthermore, a Quality Improvement Department that now oversees IPC and other patient safety issues was established.”*   That the administration recognized the need for establishing this department is perhaps the biggest victory and outcome of the HSOP surveys.  Finally, in agreeing with the reviewers comment on the length of time, I draw the reviewer to a paper (“Cluster-randomized, crossover trial of the efficacy of plain soap and water versus alcohol-based rub for surgical hand preparation in a rural hospital in Kenya”) from 2007, when we did not have an IPC nurse. This journey has been subtly reported, because of the need for an economy of words. | Please see table 3, under a new column “**Changes effected**” |
| Finally, while the authors present data only from Kijabe Hospital in Kenya, and state that their “results may not be generalizable outside KH”, they claim that “this study demonstrates that patient safety ideals are embraced in sub-Sahara Africa.” | This observation is correct. This however, is our rationale: for an area of patient care that is largely overlooked, we would like to use our institution as a yardstick for others around us.  AIC Kijabe Hospital is a tertiary institution in rural Kenya. The hospital is funded by patient fees (currently mostly the public/ government insurance), with no government support. Because of the financial background (limited), and setting (rural), our experience is a story that we hope other institutions in similar settings will look at and desire to engage in patient safety. | No changes. |
| Discussion of specific areas for improvement  Major issues:  1) [page 10/53] The authors mention that: “In the week before the questionnaire was distributed, staff were taught the precepts of patient safety, and shown the African SUSP patient safety video [44].” I might have read this wrong, however paper eludes that this was done consistently across all 4 survey time points. Even so, this activity could be considered as priming the survey participants and could likely bias the survey results regarding safety culture. | Thank you for these comments. The following statement explains our use of the HSOPS tool.  “*The authors used the HSOPS tool to establish the baseline status of patient safety culture, to raise staff awareness about patient safety and thereafter, to examine trends in patient safety culture change over time, and to evaluate the institutional cultural impact of patient safety initiatives and interventions*.”  An important part of this exercise was to get reasonable response rates. During the 4 survey administration points, staff ‘under 1 year’ of employment constituted between 20 and 40% of respondents. Educating the staff on the goals of the survey, including the importance of reporting individual perceptions without fear of any repurcussions (anonymity), was an important part of growing the safety culture.  While bias cannot be denied, we have no reason to believe that any such bias substantially affected the data collected. | In the week before the questionnaire was distributed, the SUSP core team met staff in their departments and taught them about the HSOPS tool, during these sessions, staff were taught the precepts of patient safety, and shown the African SUSP patient safety video |
| 2) [page 11/53] The authors mention that: “The team developed themes from the comments; these were then coded.” It is not very clear to follow how the qualitative analysis was done by the aforementioned statement. Usually, qualitative data is coded, and themes arise from the codes. It could be helpful if the authors expanded a little further on their data analysis approach. | Thank you for pointing out this oversight. We have corrected the manuscript. | The team coded participant free text comments into broad categories as themes emerged. The final thematic analysis involved merging similar themes into broader categories. Thematic saturation was not anticipated because of the small number of participants that wrote free text comments. |
| 3) [page 13/53] “Eleven major themes emerged from an analysis of the free comments from the 2019 survey (Table 3, Figure 1).” It is unclear whether the 2013, 2015, and 2017 surveys allowed participants to share their comments or whether only the 2019 survey included it and therefore the authors only report on qualitative aspects of the study for 2019. It could be helpful if the authors could clarify this aspect. | We have added this statement to clarify this point. Thank you | Participants in the 2013 to 2017 HSOPS responses did not write any comments in the free text space provided. |
| 4) The authors state that “The initial survey helped establish the concepts of a patient safety culture and raise staff awareness about patient safety” and that “All the HSOPS results were reported to and discussed with senior management; the senior management addressed some of issues that came out of each report.” Given that conversations seem to have happened based on the aforementioned statements, this study could have been very informative and valuable with a richer qualitative aspect to help explain the reported safety variables over time. Institutionalizing a safety culture is difficult to be captured solely via a survey and survey results. Instead, it could be very valuable to know how safety measures improved and the hospital activities that that entailed between survey periods. It takes effort, activities, and communication to build and institutionalize a safety culture and the paper does not add to that process of construction and institutionalization of the safety culture over time. For example, looking at Table 2a, it would be very helpful to have more insights into how hospital management supported patient safety, how mistakes led to positive changes, how staff actively improved patient safety. Such findings could be valuable, generalizable and the lessons transferable to other settings for institutionalizing hospital safety culture. If such data had been collected but not reported in this study, I would strongly recommend including them in a subsequent draft. | We appreciate this insight greatly, andn agree that the lessons learnt from this journey could be useful to others. We will start working on that data – although most of it will be narrative.  Thank you | No change |
| 5) The authors mention difficult working conditions that affected the 2017 results. While qualitative aspects between 2013-2015 or 2017-2019 could provide insights into building a culture of safety given the improved survey results, the 2015-2017 results could be powerful to gain insights into how difficult working conditions affect safety culture and safety outcomes via a more in-depth qualitative approach and further support the authors hypothesis about the relationship between just and safety cultures. If such data had been collected but not reported in this study, I would strongly recommend including them in a subsequent draft as well. | 2017 was a very difficult year for our institution. After the leadership change, an institution-wide culture-change pocess took place, with the hiring of change experts and indepth interviews.  We will try and se if these can be gathered together to form a paper. Thank you | No change |
| 6) [page 12/53] It is important to survey different cadres as to their perceptions of safety and I appreciate the efforts of the authors to cover diverse cadres in the hospital. However, the wide range of cadres surveyed may dilute more pronounced results from cadres that are more directly involved with safety. Also, the cadre labelled as “others” seems to combine very different sub-cadres – as per the authors, the category “others” included “Infection Prevention and Control staff, medical records, housekeeping, and security personnel” – infection staff and security personnel could have very different perspectives of the safety culture and activities based on their proximity to patients and processes in the hospital. | This view is correct. We however, followed the AHRQ definitions. Further, to ensure some degree of agreement on patient safety concepts, we held departmental meeting prior to the survey to address this issue.  Like the reviewer, we appreciate the diversity that this brings, but would argue that the benefits of getting every employee on board the safety culture is important as it glues the staff together, with a focus on |  |
| 7) The authors seem to be presenting contradicting statements and a clearer position could be helpful. The authors state that “this study demonstrates that patient safety ideals are embraced in sub-Sahara Africa” (pages 1, 5, 16) however, they are presenting data on only one hospital in Kenya, and the authors themselves state that “our results may not be generalizable outside KH” (page 16/53). | Thank you.  This statement now includes , ‘can’. | … this study demonstrates that patient safety ideals can be developed and embraced in sub-Sahara Africa, …. |
| Minor/other issues:  8) The authors state that they “performed a narrative review of literature on patient safety in sub-Sahara Africa between January 2000 and December 2020.” The authors are providing the context and engaging with the literature to situate their study. I am not sure that this review of the literature needs to be framed as a “narrative review of the literature.”  and correcting errors of that nature (e.g., use of acronyms without being explicitly defined, use of singular/plural (“chi-square tests was used”). | This has been corrected to read as shown in the statement.  We have corrected acronyms WHO and HCWs as shown  The grammar has been corrected to read, ‘were’ | The authors reviewed literature, on patient safety in sub-Sahara Africa between January 2000 and December 2020.  The World Health Organization (WHO)  Changed HCWs to healthcare workers.  …. were... |
| 9) The authors report that “the average response rate over the study period was 84.5%.” I am not sure how useful this is for the abstract of the paper. Perhaps the authors could consider providing a range for the response rate across the four different surveys over the 7-year period, like they do in the main body of the paper, instead of an average. | This is noted, and change effected. | … ranged between range 65.1% and 93.6%. |
| 10) Table 2a is somewhat cumbersome to read - perhaps the results could be re-arranged so that the statistics appear after each column (2013-2015, 2015-2017, 2017-2019). In addition, the authors could indicate significant results with an asterisk or highlight the significant results on the table in an alternative way for better visibility. | Thank you for this insight | Table 2a has been re-arranged |
| 11) As PLOS Global Health does not copyedit accepted manuscripts, I highly recommend going through the manuscript | We trully appreciate these val;uable comments |  |
