# Supplementary material for: Patient safety in a rural sub-Saharan Africa hospital: A 7-year experience at the AIC Kijabe Hospital, Kenya
Source: PLOS Glob Public Health. 2024 Nov 12;4(11):e0003919. doi: 10.1371/journal.pgph.0003919 (PMC11556696; doi:10.1371/journal.pgph.0003919)
Supplement: Attachment — Submitted filename: Response to Reviewer Comments.docx [file pgph.0003919.s006.docx]

| We only received Journal Requirements | | |
| --- | --- | --- |
| **Journal Requirement** | **Author Comment** | **Manuscript Revisions** |
| Please review your reference list to ensure that it is complete and correct. | References were corrected. Two references after changes referred to the same web page, and were merged. No new references, and no retracted references | Corrected references detailed in the cover letter. |
| While revising your submission, please upload your figure files to the Preflight Analysis and Conversion Engine (PACE) digital diagnostic tool. | Thisi was done | Uploaded PACE formatted figures |
|  |  |  |
